# Sensitivity to White Matter fMRI Activation Increases with Field Strength

**DOI:** 10.1371/journal.pone.0058130

**Published:** 2013-03-04

**Authors:** Erin L. Mazerolle, Jodie R. Gawryluk, Kim N. H. Dillen, Steven A. Patterson, Kirk W. Feindel, Steven D. Beyea, M. Tynan R Stevens, Aaron J. Newman, Matthias H. Schmidt, Ryan C.N. D’Arcy

**Affiliations:** 1 Institute for Biodiagnostics (Atlantic), National Research Council, Halifax, Nova Scotia, Canada; 2 Department of Psychology & Neuroscience, Dalhousie University, Halifax, Nova Scotia, Canada; 3 Cognitive Neuroscience, Institute of Neuroscience and Medicine, Research Centre Juelich, Juelich, Germany; 4 Department of Physics, Dalhousie University, Halifax, Nova Scotia, Canada; 5 School of Biomedical Engineering, Dalhousie University, Halifax, Nova Scotia, Canada; 6 Department of Pediatric Neurology, IWK Health Centre, Halifax, Nova Scotia, Canada; 7 Department of Radiology, Dalhousie University, Halifax, Nova Scotia, Canada; 8 Department of Anatomy and Neurobiology, Dalhousie University, Halifax, Nova Scotia, Canada; University of Minnesota, United States of America

## Abstract

Functional magnetic resonance imaging (fMRI) activation in white matter is controversial. Given that many of the studies that report fMRI activation in white matter used high field MRI systems, we investigated the field strength dependence of sensitivity to white matter fMRI activation. In addition, we evaluated the temporal signal to noise ratio (tSNR) of the different tissue types as a function of field strength. Data were acquired during a motor task (finger tapping) at 1.5 T and 4 T. Group and individual level activation results were considered in both the sensorimotor cortex and the posterior limb of the internal capsule. We found that sensitivity increases associated with field strength were greater for white matter than gray matter. The analysis of tSNR suggested that white matter might be less susceptible to increases in physiological noise related to increased field strength. We therefore conclude that high field MRI may be particularly advantageous for fMRI studies aimed at investigating activation in both gray and white matter.

## Introduction

Despite recent reports of white matter functional magnetic resonance imaging (fMRI) activation [1]–[15], the ability to detect fMRI signal changes in white matter remains controversial [15], [16]. White matter fMRI activation may be smaller in magnitude than gray matter activation [6], [12], [14], which could make detection more difficult. In fact, many of the studies reporting white matter fMRI activation have used 3 T or 4 T magnets [1], [4]–[8], [13], [14]. For gray matter, high field MRI offers inherently increased sensitivity to fMRI activation [17]–[31]. Increased field strength improves sensitivity to fMRI activation in two ways. First, the signal to noise ratio (SNR) is proportional to field strength [32], [33]. Second, the strength of the magnetic field perturbations produced by susceptibility differences is proportional to the strength of the applied field. In this way, blood oxygen level dependent (BOLD) contrast (which depends on susceptibility differences caused by the relative concentration of diamagnetic oxygenated hemoglobin and paramagnetic deoxygenated hemoglobin) is improved at higher magnetic fields [17], [32]. Increased fMRI contrast allows for improved sensitivity to brain activity (i.e., higher *z*-scores). There is considerable experimental evidence of field strength dependent sensitivity increases in gray matter [18], [19], [21]–[27], [30], [31], [34]. However, due to the widespread availability of clinical MRI systems, a large proportion of fMRI studies have historically been performed at 1.5 T [22], [23], [25], which may contribute to the overall scarcity of reports of white matter fMRI activation.

To evaluate the effect of field strength on sensitivity to white matter fMRI activation, we compared white matter activation during a finger tapping task at 1.5 T and 4 T. A similar task has previously been used to elicit white matter fMRI activation in the internal capsule at 4 T [7]. In addition to activation results, we also examined temporal signal to noise ratio (tSNR) as a function of tissue type and field strength, as this metric has been linked to successful detection of fMRI activation (e.g., [35]).

## Methods

### Ethics Statement

The research protocol was approved by the National Research Council’s Ottawa Research Ethics Board and the Capital District Health Authority Research Ethics Board. Each participant provided written informed consent prior to participation and received compensation for participating.

### Participants

Data from seven healthy participants (four females) were analyzed. The mean age (± standard deviation) of the participants was 24.5±3.5 years. All participants were right handed as assessed by the Edinburgh Handedness Inventory [36], and had normal or corrected-to-normal vision.

### Task

The task was based on a previous study in which internal capsule and sensorimotor cortex activation was observed at 4 T [7]. E-Prime (Psychology Software Tools, Inc.) was used to present stimuli, which were back-projected onto a screen mounted inside the magnet bore, and viewed through a mirror mounted on the head coil. Participants performed eight blocks of visually cued sequential finger tapping. Finger tapping was performed such that the participant used his or her thumb to touch each of the fingers in sequence. Specifically, the participant viewed black circle outlines on a white background. The circles were numbered one to four, and the participant was instructed that each circle represented a finger (e.g., “1”  = index finger). The circles were filled with red briefly and sequentially to indicate which finger the participant should touch with his or her thumb. Participants tapped their fingers at a frequency of 2 Hz. The participant performed the tapping for the right and left separately (four blocks of each hand). The hand to be used for the tapping in a given block was presented on the screen immediately before the beginning of the block (“LEFT HAND” or “RIGHT HAND”). Hand order was pseudo-random, with no more than two blocks of the same hand in a row. Each tapping block was 20 s in duration, interleaved with 20 s rest blocks.

### MRI Acquisition

All participants were scanned at both 1.5 T and 4 T. The 1.5 T MRI was a General Electric system with an eight channel head coil. The 4 T MRI used an Oxford magnet, with gradients provided by a body coil (Tesla Engineering Ltd.), a Varian INOVA console, and a transverse electromagnetic (TEM) head coil for transmit/receive (Bioengineering Inc.).

Scan order was randomized across participants. Participants 2, 3, 4, and 5 were scanned in the 1.5 T first. Both scans took place within one week for all participants. Whole-brain functional images were collected with a T2*-weighted, single-shot spiral out sequence with the following parameters: TR = 2 s, flip angle = 90°, field of view = 240×240 mm^2^, 64×64 matrix, and 22 5 mm slices with a 0.5 mm slice gap. An echo time of 40 ms was used at 1.5 T, and an echo time of 15 ms was used at 4 T to compensate for the shorter T2* at higher fields. A high resolution T1-weighted anatomic image was collected at each session for registration purposes (TR/TE = 25/5 ms, flip angle = 40°, field of view = 240×240 mm^2^, 256×256 matrix, 64 3 mm slices, no gap) using a spoiled-GRASS (SPGR) sequence at 1.5 T and a magnetization prepared fast low angle shot (MPFLASH) sequence at 4 T.

### Data Analysis

#### Pre-Processing and statistical analyses

Pre-processing and statistical analyses were performed with the fMRI expert analysis tool (FEAT) version 5.98 in FMRIB Software Library (FSL; [37]). Pre-statistics processing included the following steps: motion correction using MCFLIRT [38], non-brain removal using BET [39], spatial smoothing using a Gaussian kernel (5 mm full width at half maximum), mean-based intensity normalisation, and highpass temporal filtering (Gaussian weighted least-squares straight line fitting, with σ = 50 s). The analysis employing these pre-processing steps is referred to as the smoothed analysis. In addition, a second pre-processing pipeline was tested, in which spatial and temporal filtering were excluded to evaluate the contributions of these steps on white matter fMRI activation (unsmoothed analysis).

For the first-level analyses, time series statistical analyses were carried out using FMRIB’s Improved Linear Model (FILM) with local autocorrelation correction [40]. Motion parameters (output from the motion correction) were included in the model as regressors of no interest. Activation was modelled by convolving the double gamma hemodynamic response function with boxcar functions representing the task (one each for left and right hand tapping) with in FEAT. *Z* (Gaussianised *t*) statistic images were reported using a threshold for clusters determined by *z*>2.3 and a (corrected) cluster significance threshold of *p*<0.05 [41]. *T*-contrasts were calculated to evaluate activation for left hand tapping, right hand tapping, and the combination of left and right hand tapping. FLIRT was used to register the functional images to the anatomic images (seven degrees of freedom [DOF]), and to register the anatomic images to the Montreal Neurological Institute template (12 DOF; [38], [42]). Registration to standard space was then further refined using FNIRT nonlinear registration [43], [44]. The non-linear registration approach was selected for two reasons: 1) FNIRT is commonly used for white matter in DTI studies [45]; and 2) FNIRT has been shown to result in improved registration results for subcortical structures [46], [47]. The results of the registration algorithms were visually inspected to ensure adequate performance. Group level activation results for 1.5 T, 4 T, 1.5 T>4 T, and 4 T>1.5 T were examined using FMRIB’s Local Analysis of Mixed Effects stage 1 (FLAME 1; [48]–[50]).

#### Region of interest analysis

**Figure 1:**
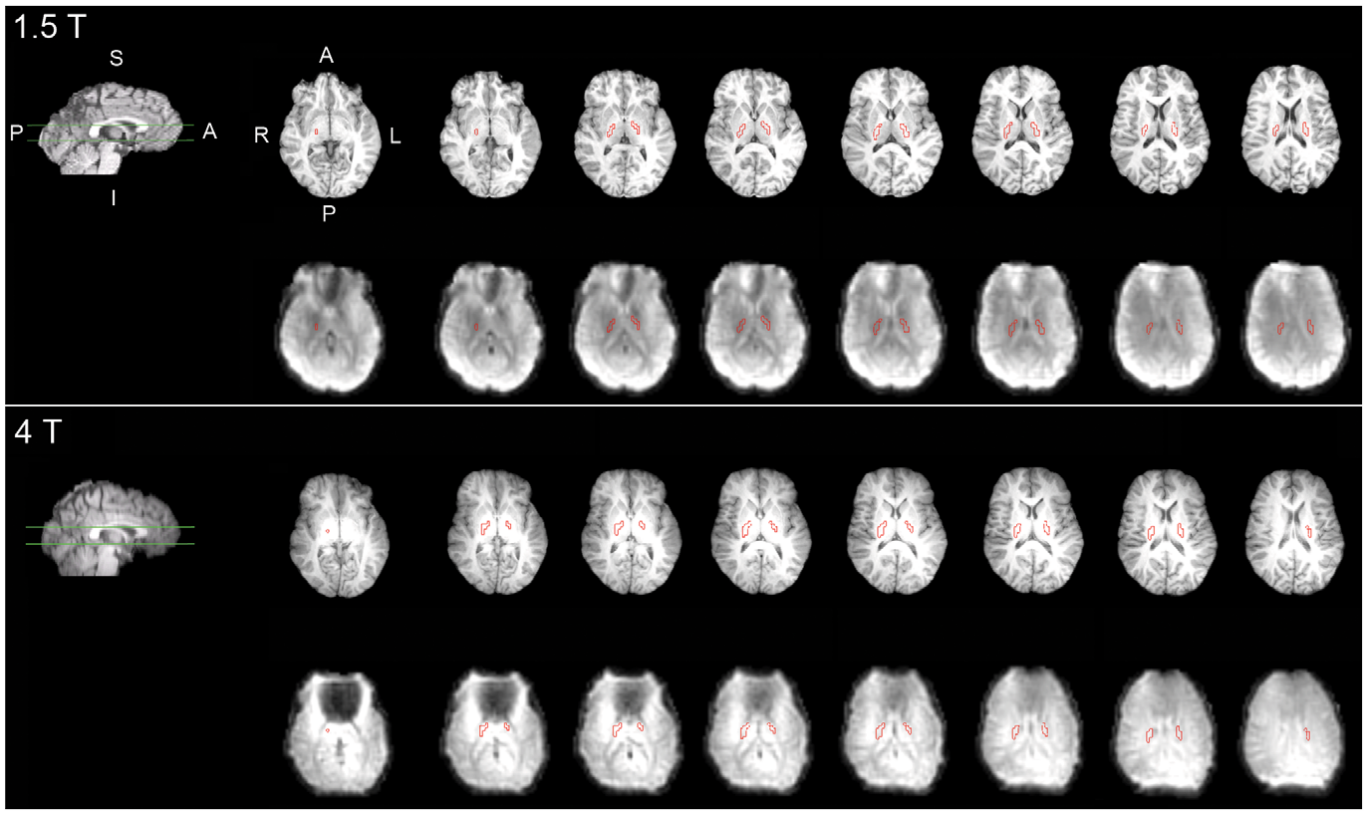
Outline of PLIC ROI (red) overlaid on a functional volume and the corresponding slices of the high resolution anatomic image for a representative participant. Top: 1.5 T; bottom: 4 T. The green lines on the sagittal images depict the inferior-superior range of the subsequent axial slices. S: superior; I: inferior; A: anterior; P: posterior; R: right; L: left. Please note that for display purposes, the functional images were upsampled (256×256×88).

At both the group and individual levels, FSL’s featquery tool was used to evaluate activation in the posterior limb of the internal capsule (PLIC). We selected the PLIC ROI *a priori* because it contains corticospinal fibres connecting the primary motor and sensory cortices with the spinal cord, and has been previously shown using combined fMRI and diffusion tensor imaging (DTI) to house the structural connections associated with finger tapping activation [51]. The region of interest (ROI) was selected from the JHU ICBM-DTI White Matter Labels Atlas [52], registered to participant space using the results of the image registrations described above, and manually verified. By manually verifying atlas-based ROIs and applying them to individual-level data, this approach greatly reduces potential gray matter contamination of the ROI that might result from registration errors. The PLIC ROIs for a representative participant, for both field strengths, are shown in Figure 1. Note that although the region of orbitofrontal susceptibility artifact signal dropout is larger for the 4 T images, the PLIC ROI does not overlap with any low-signal voxels. The PLIC ROIs are shown for each participant in Figure S1.

To determine whether activation on the white matter ROI likely originated from the PLIC (rather than, for example, resulting from gray matter signal contamination of the ROI during spatial smoothing and/or other causes of partial volume effects), we also evaluated whether the activation on the PLIC ROI included local maxima (identified using FSL’s cluster tool). To be considered a local maximum, the *z*-score of the voxel had to be the largest within an 8 mm radius. We have employed a similar approach in previous work (e.g., [6]).

In addition to investigating the PLIC ROI, we also evaluated activation in sensorimotor cortex. Sensorimotor cortex also is activated by the finger tapping task and thus serves as a region of gray matter that can be compared to the white matter activation results. The sensorimotor cortex ROI was defined as the precentral gyrus, postcentral gyrus, and the supplementary motor cortex using the Harvard-Oxford Cortical Atlas in FSL. The sensorimotor cortex ROI was further refined to ensure only gray matter was included. Gray matter masks were defined for each participant using FMRIB’s Automated Segmentation Tool (FAST; [53]). *A priori* tissue probability maps were used to initialize the segmentation. A 99% probability threshold was applied to the resulting partial volume estimate maps. For both the PLIC and the sensorimotor cortex ROIs, activation extent (number of significantly activated voxels), maximum *z*-score, and mean *z*-score and mean percent signal change of significantly activated voxels were calculated.

#### Temporal SNR analysis

Temporal SNR was calculated for fMRI data that had not been pre-processed, except for motion correction. Therefore, the data input to the tSNR analysis was pre-processed using the same steps that were applied to the data in the unsmoothed analysis. The time series were also detrended using fsl_regfilt to remove activation-related variance. Thus, the tSNR values reflect factors such as temporal drift and autocorrelation, which can impact activation sensitivity if the corrections applied in the fMRI analysis do not fully compensate for their effects. Voxel-wise tSNR was calculated as the mean of the time series divided by the standard deviation of the time series. Mean tSNR values were derived for the whole brain (conservatively masked using the 80^th^ percentile voxel intensity to remove areas of susceptibility artifact related signal dropout), and for gray and white matter separately. Gray and white matter masks were created by thresholding the partial volume estimates output by FAST at 99% probability and registering the masks to functional space (nearest neighbor interpolation). The cerebellum was excluded by registering the cerebellum mask from the Montreal Neurological Institute Structural Atlas [54], [55] to each participant. We also calculated the mean tSNR for the PLIC and sensorimotor cortex ROIs.

#### Power spectra analysis

To evaluate the potential sources of variance contributing to tissue type differences in activation and tSNR results, the power spectra of the time series data were evaluated. Pre-processed fMRI data were detrended using fsl_regfilt to remove variance associated with the model. Thus, the remaining signal variance is assumed to be noise. Voxel-wise power spectra of the noise were calculated using the fslpspec function, and averaged across participants for the gray and white matter ROIs (described above). Power differences between the ROIs were then calculated for both 1.5 T and 4 T.

## Results

### Functional MRI Activation

**Figure 2:**
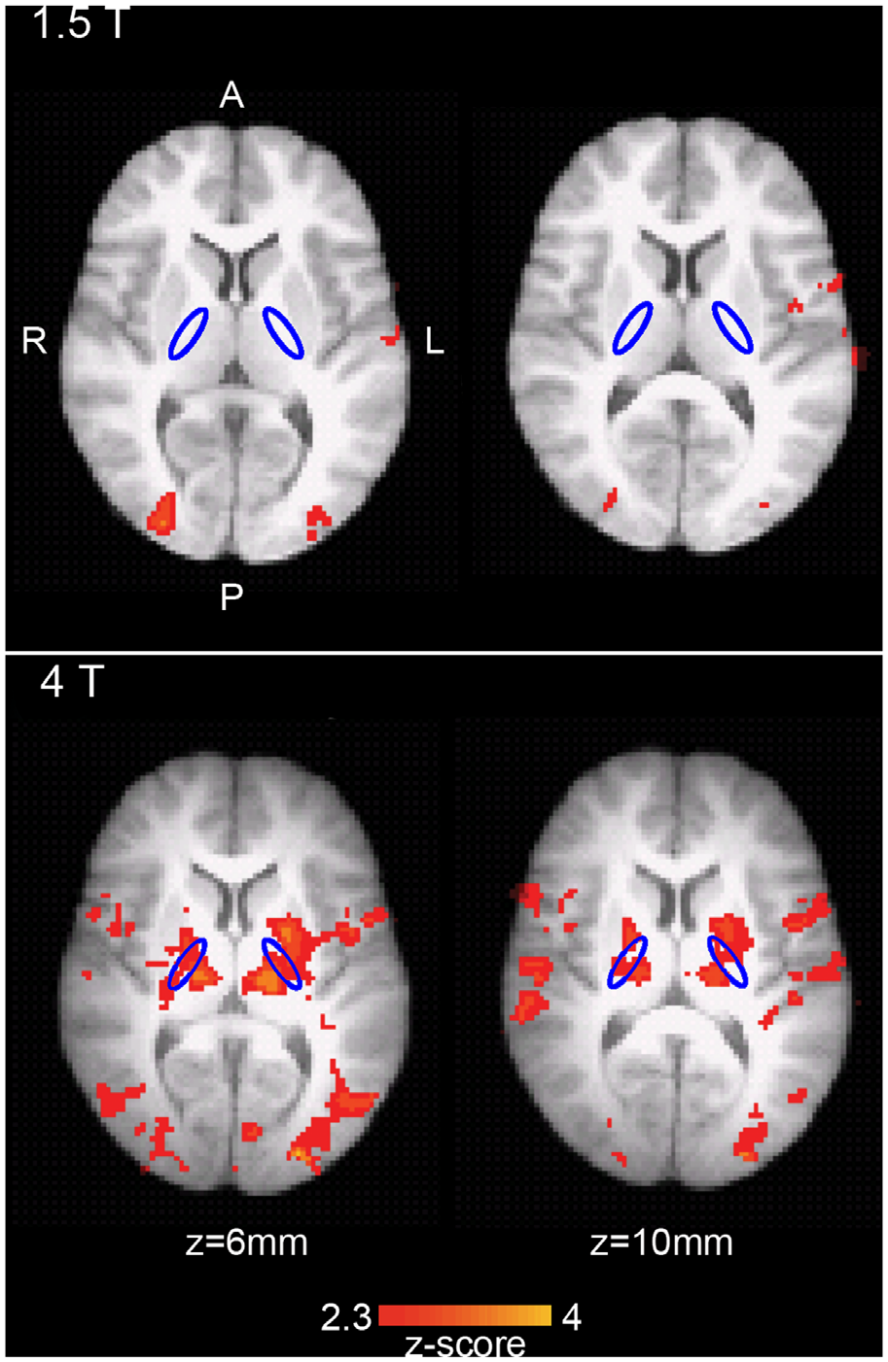
Group level activation (n = 7) in the finger tapping task (smoothed analysis). Top: 1.5 T; bottom: 4 T. Activation is overlaid on the MNI brain. Two slices through the PLIC are shown (z = 6 mm and z = 10 mm, MNI coordinates). The PLIC ROI is outlined in blue. Significant activation is shown in red-yellow (*z*>2.3, *p*<0.05). A: anterior; P: posterior; R: right; L: left.

The results presented here focus on left and right hand tapping combined for the smoothed analysis. When analyzed separately, left and right hand tapping produced the expected contralateral pattern of activation (i.e., relatively greater right hemisphere activation for left hand tapping and vice versa; see Table S1 for details). The group level smoothed analysis revealed activation in sensorimotor cortex, the supplementary motor area, and the cerebellum for both 1.5 T and 4 T. Activation was observed in the PLIC ROI for 4 T, but not 1.5 T (see Figure 2). When compared statistically using a *t*-contrast, no activation was observed in the 1.5 T>4 T contrast. For the 4 T>1.5 T contrast, significant activation was observed, including in the PLIC and motor cortex ROIs (Table 1). However, no local maximum was localized to the PLIC ROI, for either the 4 T or 4 T>1.5 T contrasts.

**Table 1:** Group level region of interest (ROI) results.

ROI	Contrast	Max *z*-score	Extent (activated voxels/total ROI voxels)	% of ROI activated
PLIC	1.5 T	0.00	0/566	0.00
	4 T	3.82	226/566	39.93
	4 T>1.5 T	2.62	13/566	2.30
Sensorimotor cortex	1.5 T	4.57	5272/25013	21.08
	4 T	5.00	9819/26609	36.90
	4 T>1.5 T	3.96	785/25007	3.14

**Figure 3:**
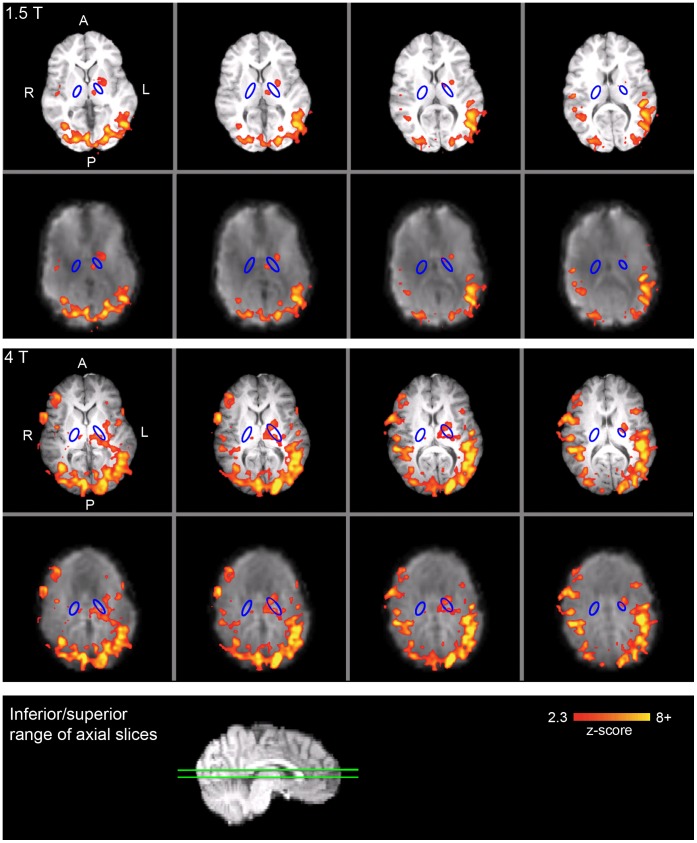
Individual level activation in the PLIC for a representative participant (smoothed analysis). Top panel: 1.5 T; middle panel: 4 T; bottom panel: sagittal image with green lines that depict the inferior-superior range of the axial slices above. For both the top and middle panels, the top row depicts the activation overlaid on the high resolution anatomic image and the bottom row depicts the activation overlaid on the functional image (registered to the high resolution anatomic image). The PLIC ROI is outlined in blue. Significant activation is shown in red-yellow (*z*>2.3, *p*<0.05). A: anterior; P: posterior; R: right; L: left.

**Figure 4:**
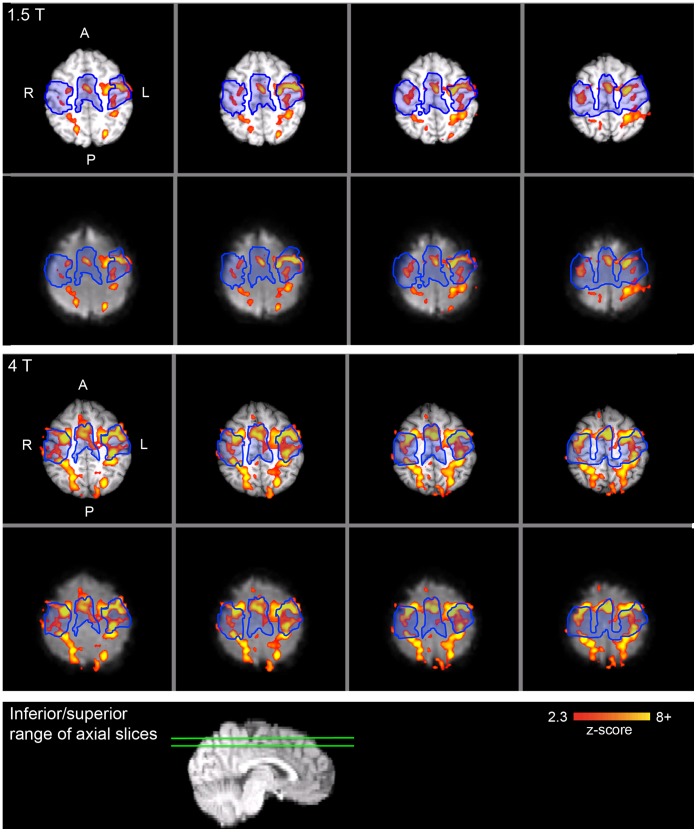
Individual level sensorimotor cortex ROI activation for a representative participant (smoothed analysis). Top panel: 1.5 T; middle panel: 4 T; bottom panel: sagittal image with green lines that depict the inferior-superior range of the axial slices above. For both the top and middle panels, the top row depicts the activation overlaid on the high resolution anatomic image and the bottom row depicts the activation overlaid on the functional image (registered to the high resolution anatomic image). The sensorimotor cortex ROI is outlined in blue. Significant activation is shown in red-yellow (*z*>2.3, *p*<0.05). A: anterior; P: posterior; R: right; L: left.

For the smoothed analysis, activation results for a representative participant are presented in Figures 3 and 4 for the PLIC and sensorimotor cortex ROIs, respectively. Individual level PLIC ROI results are summarized in Table 2, and were consistent with the group level findings. While only five of the seven participants showed PLIC activation at 1.5 T, PLIC activation was detected in all seven participants at 4 T. Furthermore, six of the seven participants had a higher maximum *z*-score and larger extent of activation in the PLIC for 4 T relative to 1.5 T. The maximum *z*-score was significantly greater for 4 T than 1.5 T (*t*(6) = 2.40, *p*<0.05, one-tailed). The extent of activation was also significantly greater for 4 T than 1.5 T (*t*(6) = 2.18, *p*<0.05, one-tailed). In addition, the mean *z*-score across significantly activated voxels was significantly greater for 4 T than 1.5 T (*t*(6) = 2.06, *p*<0.05, one-tailed); however, this finding should be interpreted with caution due to the large proportion of participants with very few activated voxels in the PLIC ROI at 1.5 T. Only one participant had a local maximum located on the PLIC ROI for 1.5 T, whereas four of the seven participants had local maxima on the PLIC ROI for 4 T.

**Table 2:** Summary statistics for the PLIC ROI (individual level, smoothed analysis).

A. 1.5 T.					
Participant	Max *z*-score	Extent (activated voxels/totalROI voxels)	% of ROI activated	Mean ± SD *z*-score(activated voxels only)	Local maxin ROI?
1	2.40	0/44	0.00	0.00	no
2	5.31	13/40	32.50	3.16±0.80	no
3	2.57	0/42	0.00	0.00	no
4	3.13	2/40	5.00	2.77±0.52	no
5	3.44	1/42	2.38	3.44±nan	yes
6	2.48	1/48	2.08	2.48±nan	no
7	3.26	5/36	13.89	2.99±0.18	no
**Average**	**3.23**	–	**7.98**	**2.12**	**1/7**
SD (across participants)	1.01	–	11.82	1.48	–
**B. 4 T.**					
**Participant**	**Max ** ***z*** **-score**	**Extent (activated voxels/total ROI voxels)**	**% of ROI activated**	**Mean ± SD ** ***z*** **-score** **(activated voxels only)**	**Local max** **in ROI?**
1	4.08	9/46	19.57	3.03±0.60	no
2	3.64	6/49	12.24	2.77±0.45	no
3	4.05	5/46	10.87	3.25±0.74	no
4	5.18	8/38	21.05	3.39±0.76	yes
5	5.10	11/47	23.40	3.60±0.90	yes
6	3.91	9/49	18.37	2.90±0.47	yes
7	4.65	21/40	52.50	3.71±0.55	yes
**Average**	**4.37**	**–**	**22.57**	**3.23**	**4/7**
SD (across participants)	0.60	–	13.96	0.36	–

The individual level sensorimotor cortex ROI results are presented in Table 3. While there was no difference in maximum *z*-score between 1.5 T and 4 T (*t*(6) = 0.85, *p* = n.s., one-tailed), the mean *z*-score of activated voxels was significantly greater for 4 T than 1.5 T (*t*(6) = 2.65, *p*<0.05, one-tailed). A larger extent of sensorimotor cortex activation was observed at 4 T versus 1.5 T (47.02±11.61% and 33.44±10.90% for 4 T and 1.5 T, respectively); this effect was statistically significant (*t*(6) = 2.43, *p*<0.05, one-tailed).

**Table 3:** Summary statistics for the sensorimotor cortex ROI (individual level, smoothed analysis).

A. 1.5 T.					
Participant	Max *z*-score	Extent (activated voxels/totalROI voxels)	% of ROI activated		Mean *z*-score(activated voxels only)
1	12.11	105/480	21.88		4.61
2	12.58	189/379	49.87		5.32
3	9.56	115/493	23.33		4.05
4	9.57	217/622	34.89		4.80
5	9.45	106/457	23.19		4.17
6	10.42	204/509	40.08		4.62
7	11.53	159/389	40.87		4.63
**Average**	**10.74**	**–**	**33.44**		**4.60**
SD	1.32	–	10.90		0.42
**B. 4 T.**					
**Participant**	**Max ** ***z*** **-score**	**Extent (activated voxels/total** **ROI voxels)**		**% of ROI activated**	**Mean ** ***z*** **-score** **(activated voxels only)**
1	11.69	285/547		52.10	5.33
2	12.23	193/427		45.20	5.64
3	9.94	193/536		36.01	4.35
4	9.68	174/586		29.69	4.47
5	12.39	255/503		50.70	5.18
6	10.30	307/470		65.32	4.97
7	11.60	187/373		50.13	5.20
**Average**	**11.12**	**–**		**47.02**	**5.02**
SD	1.12	–		11.61	0.46

For completeness, we have included mean percent signal change for the ROIs and field strengths in Table S2. Note that due to the choice of TE at each field strength (40 ms for 1.5 T, 15 ms for 4 T), percent signal change would be expected to be similar for 1.5 T and 4 T results.

Overall, the same pattern of white matter fMRI results was observed for unsmoothed data, with PLIC activation in 43% of participants at 1.5 T, versus 100% of participants at 4 T. The individual level PLIC ROI results for the unsmoothed analysis can be found in Table S3.

### Temporal SNR

**Figure 5:**
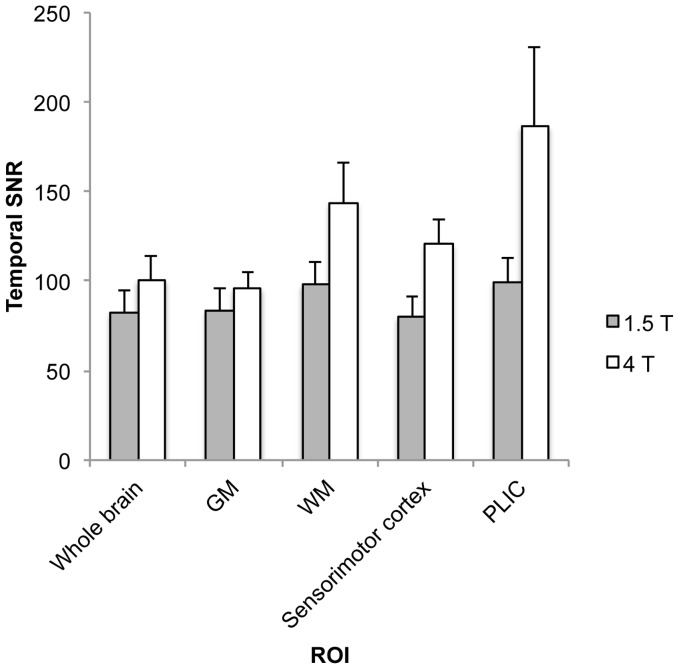
ROI analysis of grand average (n = 7) temporal SNR results. Error bars represent one standard deviation.

Temporal SNR results are presented in Figure 5. The mean tSNR for the whole brain was 82.4 for 1.5 T and 100.6 for 4 T (i.e., an increase of 22.1% for 4 T). Whole brain tSNR was significantly greater at 4 T relative to 1.5 T (*t*(6) = 3.52, *p*<0.01, one-tailed). The gray matter tSNR was 83.5 and 96.0 for 1.5 T and 4 T, respectively (i.e., an increase of 15.0% for 4 T), whereas the white matter tSNR was 97.8 and 144.0 for 1.5 T and 4 T, respectively (i.e., an increase of 47.2% for 4 T). The tSNR difference between white matter and gray matter was significantly greater at 4 T than 1.5 T (*t*(6) = 5.69, *p*<0.001, one-tailed). The relatively larger field strength dependent tSNR increase for white matter was also observed when comparing the PLIC and sensorimotor cortex ROIs. For the sensorimotor cortex ROI, tSNR was 82.8 and 119.7 for 1.5 T and 4 T, respectively (i.e., an increase of 44.6% for 4 T). For the PLIC ROI, tSNR was 99.5 and 186.3 for 1.5 T and 4 T, respectively (i.e., an increase of 87.2% for 4 T). The tSNR difference between the PLIC and sensorimotor cortex ROIs was significantly greater at 4 T than 1.5 T (*t*(6) = 2.83, *p*<0.05, one-tailed).

### Power Spectra

**Figure 6:**
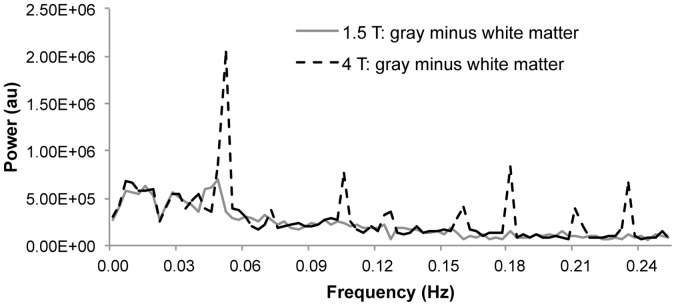
Grand average (n = 7) power spectra difference between gray and white matter. Note that the signals have been detrended to remove activation-related variance; thus the power spectra are assumed to represent the frequency components of the noise.

To evaluate the potential sources of tissue type differences in terms of activation and tSNR results, the power spectra of the noise in the gray and white matter ROIs were evaluated for both 1.5 T and 4 T. The difference spectra (gray matter minus white matter) are depicted in Figure 6. At 1.5 T, the tissue type differences are relatively uniform across frequencies. In contrast, a large peak difference at approximately 0.05 Hz is apparent at 4 T, as well as a number of smaller peak differences at higher frequencies, indicating increased power at these frequencies for gray matter at 4 T. As discussed below, physiological noise is often aliased to low frequencies such as 0.05 Hz at typical TRs [56].

## Discussion

### Summary of Findings

Consistent with previous results, PLIC activation was detected at 4 T for a finger tapping task [7]. As predicted, group level sensitivity to PLIC activation was increased at 4 T relative to 1.5 T. In addition, PLIC activation was detected in more individuals at 4 T than 1.5 T. Furthermore, this pattern of results was the same even when no smoothing was employed. At conventional thresholds, 1.5 T may not yield enough sensitivity to detect fMRI signal changes in white matter. Furthermore, we found relatively greater field strength dependent tSNR gains for white matter relative to gray matter, suggesting that high field may be particularly advantageous for white matter fMRI. We speculate on the possible mechanisms of this phenomenon in the *Effect of Physiological Noise* section below.

We determined whether local activation maxima were observed on the PLIC ROI to evaluate if the activation on the white matter ROI likely originated from the PLIC rather than from gray matter signal contamination (see below for a discussion of partial volume effects). The individual level analysis revealed local maxima on the PLIC ROI for the majority of participants at 4 T. However, no local maxima were observed on the PLIC ROI for the group analysis. Individual differences in the functional organization of the PLIC may have contributed variance such that a PLIC local maximum could not be observed on the group activation maps, but more research is needed with larger sample sizes to fully interpret this finding.

At the group level, statistical contrasts revealed that 4 T fMRI was significantly more sensitive to motor cortex activation (Table 1). At the individual level, greater activation extent and greater mean *z*-scores were observed for 4 T relative to 1.5 T in gray matter (Table 3), which is consistent with previous studies of the field strength dependence of BOLD fMRI [17]–[27], [29]–[31].

Consistent with previous findings, we observed greater tSNR for white matter than gray matter (Figure 5; [35], [57]). Importantly, field strength related increases in tSNR were significantly greater for white matter than gray matter. Given that tSNR is linked to successful detection of fMRI activation (e.g., [35]), this finding is consistent with the notion that high field fMRI is particularly important for detecting activation in white matter.

### Effect of Physiological Noise

In general, physiological noise is more prominent at higher fields due to the relationship between physiological noise and image SNR [21], [26]. Consistent with previous research demonstrating that physiological noise comprises a smaller proportion of the total noise in white matter compared to gray matter [24], [58], the spectral analysis of the noise revealed that at 4T, there was increased power in gray matter relative to white matter in the low frequency range to which physiological artifacts are often aliased (Figure 6; [56]). Given that white matter signals are less contaminated by physiological noise, white matter may be relatively more robust to field strength related increases in physiological noise than gray matter. Physiological noise differences between gray and white matter might be attributed to differences in vasculature between the tissue types [59], [60]. However, other sources of noise differences between gray and white matter cannot yet be ruled out. Future studies should employ physiological noise removal procedures to evaluate whether sensitivity at 4 T could be enhanced, particularly for gray matter activation.

### Caveats

#### Susceptibility induced field gradients at 4 T

In the 4 T data, the close proximity of the inferior parts of the PLIC ROI to regions of signal dropout associated with orbitofrontal susceptibility artifact may have affected the activation results. For example, the image distortions at the boundaries of the dropout region may have contaminated the PLIC ROI. If so, task correlated signal intensity changes in the distorted regions (i.e., task correlated motion) may have been erroneously characterized as activation. However, we included the estimated motion parameters (output from the motion correction) as regressors of no interest in our activation model, which reduces the potential contribution of movement-related signal changes that survive the motion correction [61].

#### Spatial resolution and partial volume effects

At the current nominal spatial resolution of the fMRI data (3.75×3.75×5 mm^3^), the dimensions of the PLIC (less than 10 mm in the left-right dimension on most slices; Oishi et al., 2005) are such that partial volume effects likely exist in some voxels in the PLIC ROI. In this study, we were able to evaluate whether the fMRI activation likely originated in the PLIC ROI (as opposed to neighbouring gray matter regions) by determining whether an activation local maximum was localized to the white matter region. At 4 T, the majority of participants had local maxima co-localized to the PLIC ROI, providing evidence that the activated voxels in the PLIC were not merely the result of partial volume effects. Furthermore, by repeating the statistical analysis on unsmoothed data, we also demonstrated that the white matter fMRI activation does not result from partial volume effects introduced during data processing. In the future, higher resolution fMRI scans will be crucial to disentangling the signal changes associated with the different tissue types. However, higher spatial resolution is associated with lower SNR. As we have demonstrated in the current study, white matter fMRI activation may be difficult to detect at lower SNR, which could limit the spatial resolution for white matter fMRI research. Studies with high spatial resolution might benefit from tissue-specific smoothing, which could improve SNR without resulting in gray matter contamination of white matter signals.

#### Sample size

Only seven participants were included in this study. This is a relatively small sample; however, previous studies of field strength effects have used similar sample sizes (e.g., [24]–[26]). In addition, the within-subjects nature of the experimental design allowed an examination of the effect of field strength at the individual level to supplement the group results. We demonstrated that the pattern observed in the group results (greater sensitivity to white matter fMRI activation at high field) was also observed for the majority of participants, suggesting that the group results are representative. Nevertheless, this line of research should be continued with larger sample sizes, which may help to clarify the functional significance of the individual variability in the presence of local activation maxima in white matter (Table 2).

#### MRI systems

The MRI systems used in the current study were from different manufacturers (1.5 T GE versus 4 T Varian). Previous studies have suggested that between-subject differences are larger than differences between scanner sites/manufacturers, which provides support for the validity of comparing results from different scanner manufacturers [62], particularly when using a within-subjects design such as the approach we employed. Even within manufacturers, MRI systems of different field strengths inherently differ in terms of gradient and RF hardware, for example. These differences potentially confound studies investigating field strength effects.

#### Potential contributions of large veins

Interpreting fMRI studies requires consideration of the contribution of large veins, which can result in activated voxels that are located up to a centimetre away from the site of neural activation (e.g., [63]). Given the importance of this issue for interpreting activation results, future investigations of white matter fMRI activation should acquire venograms in order to evaluate the potential contribution of large veins.

### Conclusion

We have shown that high field fMRI may be particularly important for detecting activation in white matter, given that field strength dependent increases in tSNR and fMRI sensitivity may be greater for white matter than for gray matter. The scarcity of reports of white matter fMRI activation (relative to gray matter activation) may be due in part to the prevalence of 1.5 T MRI systems.

## Supporting Information

Figure S1 **A:** PLIC ROI overlaid on a functional volume from each participant (1.5 T data). **B:** PLIC ROI overlaid on a functional volume from each participant (4 T data).
(PDF)

Table S1 Group level region of interest (ROI) results for left and right finger tapping (smoothed analysis).
(DOCX)

Table S2 Mean percent signal change for significantly activated voxels (smoothed analysis).
(DOCX)

Table S3 Summary statistics for the PLIC ROI (individual level, unsmoothed analysis).
(DOCX)
