# Supplementary material for: Sensitivity to White Matter fMRI Activation Increases with Field Strength
Source: PLoS One. 2013 Mar 4;8(3):e58130. doi: 10.1371/journal.pone.0058130 (PMC3587428; doi:10.1371/journal.pone.0058130)
Supplement: Figure S1 — A: PLIC ROI overlaid on a functional volume from each participant (1.5 T data). B: PLIC ROI overlaid on a functional volume from each participant (4 T data). (PDF) [file pone.0058130.s001.pdf]

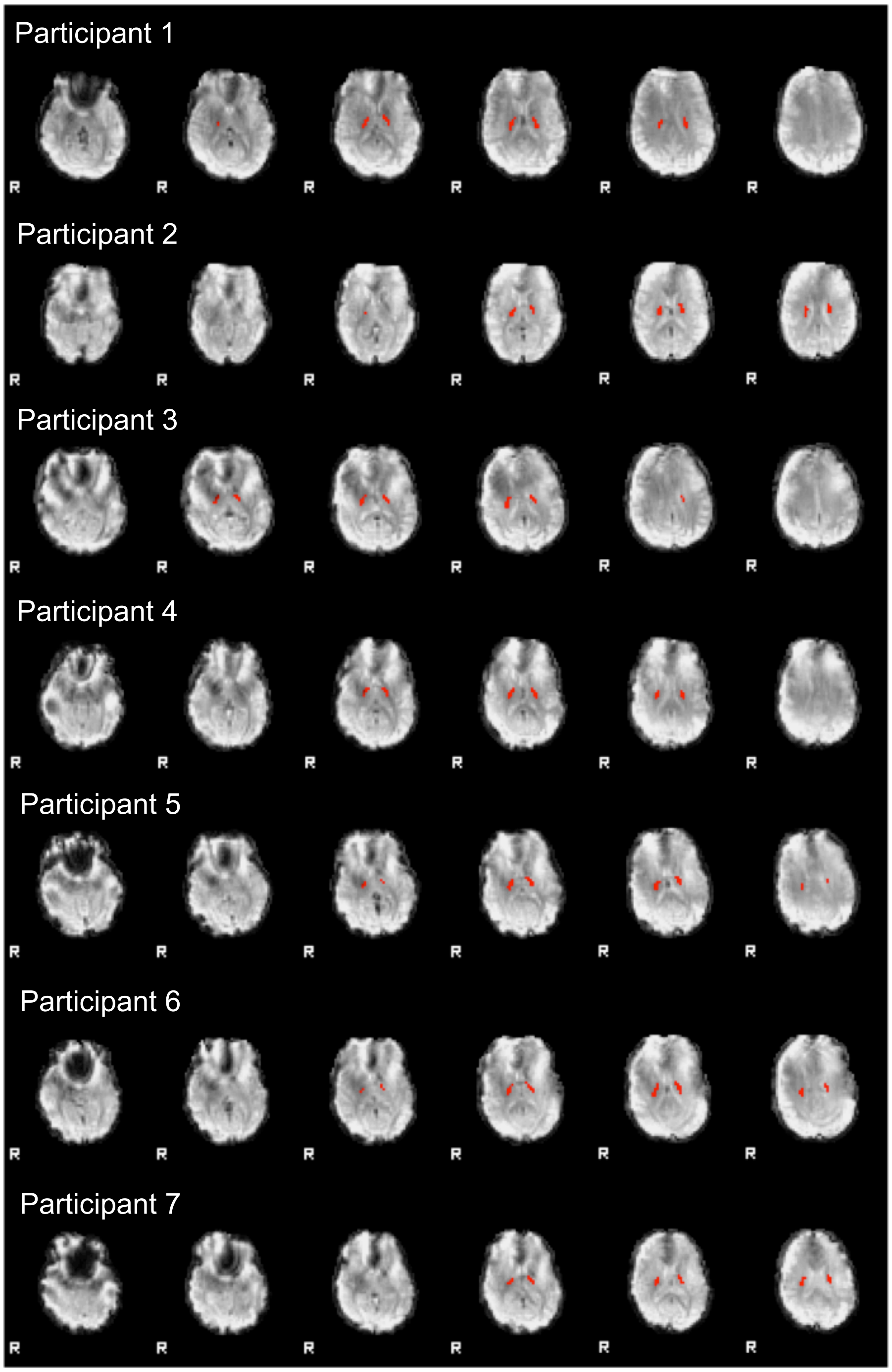

Figure S1. A: PLIC ROI overlaid on a functional volume from each participant (1.5 T data).

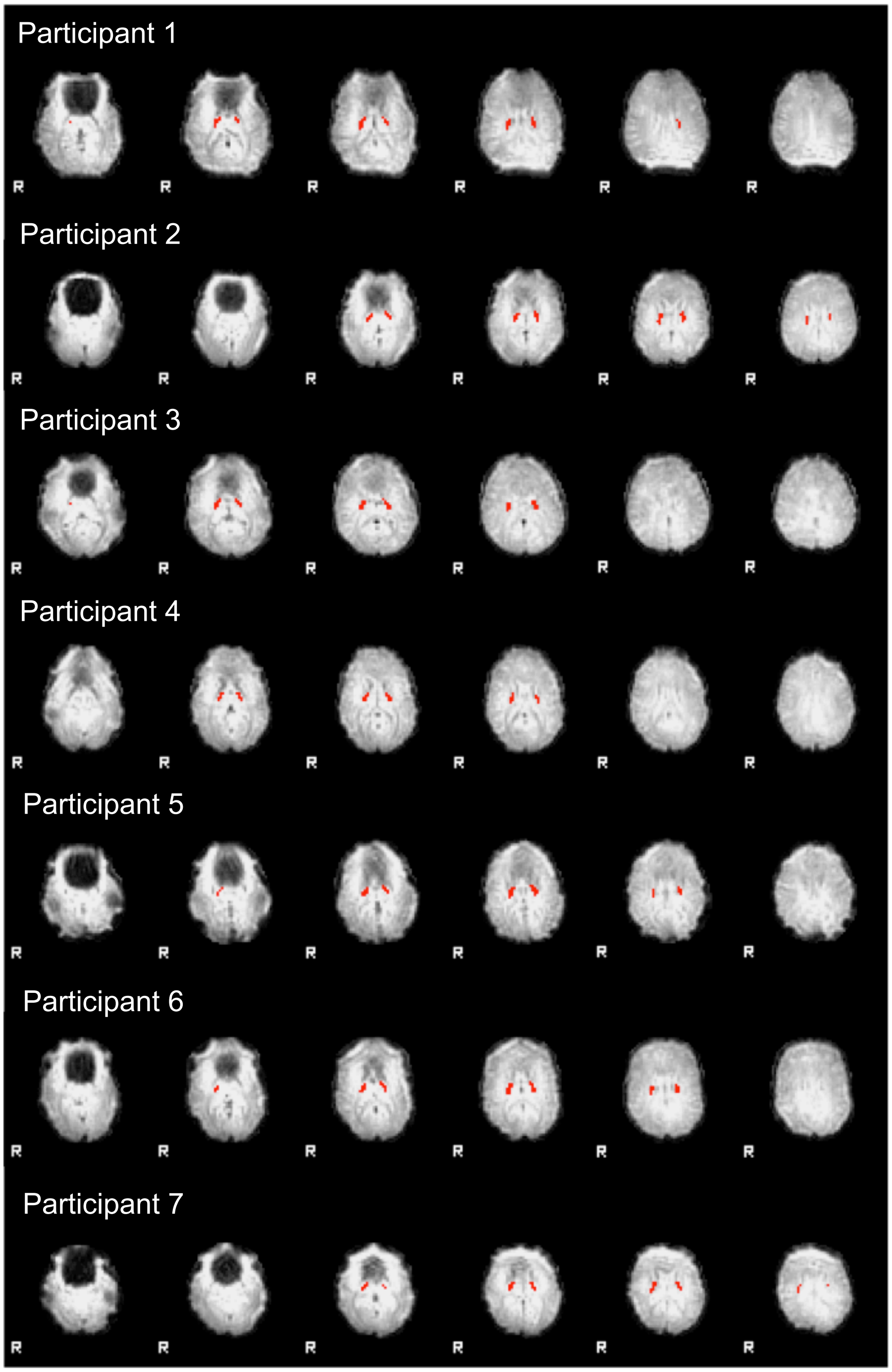

Figure S1. B: PLIC ROI overlaid on a functional volume from each participant (4 T data).
