# Supplementary material for: Sensitivity to White Matter fMRI Activation Increases with Field Strength
Source: PLoS One. 2013 Mar 4;8(3):e58130. doi: 10.1371/journal.pone.0058130 (PMC3587428; doi:10.1371/journal.pone.0058130)
Supplement: Table S1 — Group level region of interest (ROI) results for left and right finger tapping (smoothed analysis). (DOCX) [file pone.0058130.s002.docx]

| **Table S1.** Group level region of interest (ROI) results for left and right finger tapping (smoothed analysis). | | | | | |
| --- | --- | --- | --- | --- | --- |
| **Field strength** | **ROI** | **Condition** | **Max z-score** | **Extent (activated voxels/total ROI voxels)** | **% of ROI activated** |
| 1.5 T | Left Sensorimotor | Left tapping | 4.10 | 2063/12962 | 15.92 |
|  |  | Right tapping | 4.49 | 3719/12962 | 28.69 |
|  | Right Sensorimotor | Left tapping | 3.58 | 1135/12271 | 9.25 |
|  |  | Right tapping | 3.42 | 248/12271 | 2.02 |
| 4 T | Left Sensorimotor | Left tapping | 4.70 | 2542/13735 | 18.51 |
|  |  | Right tapping | 5.29 | 6100/13735 | 44.41 |
|  | Right Sensorimotor | Left tapping | 5.00 | 4864/12874 | 37.82 |
|  |  | Right tapping | 4.40 | 1878/12874 | 14.20 |
| 1.5 T | Left PLIC | Left tapping | - | 0/263 | 0 |
|  |  | Right tapping | - | 0/263 | 0 |
|  | Right PLIC | Left tapping | - | 0/303 | 0 |
|  |  | Right tapping | - | 0/303 | 0 |
| 4 T | Left PLIC | Left tapping | - | 0/263 | 0 |
|  |  | Right tapping | 3.69 | 186/263 | 70.72 |
|  | Right PLIC | Left tapping | 3.19 | 159/303 | 52.48 |
|  |  | Right tapping | 2.31 | 1/303 | 0.01 |
