# Supplementary material for: Sensitivity to White Matter fMRI Activation Increases with Field Strength
Source: PLoS One. 2013 Mar 4;8(3):e58130. doi: 10.1371/journal.pone.0058130 (PMC3587428; doi:10.1371/journal.pone.0058130)
Supplement: Table S2 — Mean percent signal change for significantly activated voxels (smoothed analysis). (DOCX) [file pone.0058130.s003.docx]

Table S2. Mean percent signal change for significantly activated voxels (smoothed analysis).

|  | **Sensorimotor cortex ROI** | | **PLIC ROI** | |
| --- | --- | --- | --- | --- |
| **Participant** | **1.5 T** | **4 T** | **1.5 T** | **4 T** |
| 1 | 1.60 | 1.25 | 0.00 | 0.33 |
| 2 | 1.58 | 0.91 | 0.51 | 0.18 |
| 3 | 1.42 | 0.99 | 0.00 | 0.42 |
| 4 | 1.17 | 0.71 | 0.41 | 0.22 |
| 5 | 1.31 | 1.38 | 0.59 | 0.42 |
| 6 | 1.32 | 1.13 | 0.51 | 0.24 |
| 7 | 1.68 | 1.43 | 0.47 | 0.54 |
