# Supplementary material for: Sensitivity to White Matter fMRI Activation Increases with Field Strength
Source: PLoS One. 2013 Mar 4;8(3):e58130. doi: 10.1371/journal.pone.0058130 (PMC3587428; doi:10.1371/journal.pone.0058130)
Supplement: Table S3 — Summary statistics for the PLIC ROI (individual level, unsmoothed analysis). (DOCX) [file pone.0058130.s004.docx]

**Table S3**. Summary statistics for the PLIC ROI (individual level, unsmoothed analysis).

| **A. 1.5 T.** | | | | | |
| --- | --- | --- | --- | --- | --- |
| **Participant** | **Max *z*-score** | **Extent (activated voxels/total ROI voxels)** | **% of ROI activated** | **Mean ± SD *z*-score (activated voxels only)** | **Local max in ROI?** |
| 1 | - | 0/44 | 0.00 | N/A | no |
| 2 | 3.45 | 3/40 | 7.50 | 2.88±0.49 | no |
| 3 | - | 0/42 | 0.00 | N/A | no |
| 4 | - | 0/40 | 0.00 | N/A | no |
| 5 | - | 0/42 | 0.00 | N/A | no |
| 6 | 3.72 | 5/48 | 10.42 | 2.86±0.52 | yes |
| 7 | 2.45 | 1/36 | 2.78 | 2.45±nan | no |
| **Average** | **3.21** | **-** | **6.90** | **2.73** | **-** |
| SD (across participants) | 0.67 | - | 3.85 | 0.24 | - |
|  |  |  |  |  |  |
| **B. 4 T.** | | | | | |
| **Participant** | **Max *z*-score** | **Extent (activated voxels/total ROI voxels)** | **% of ROI activated** | **Mean ± SD** ***z*-score (activated voxels only)** | **Local max in ROI?** |
| 1 | 2.95 | 4/46 | 8.70 | 2.65±0.25 | no |
| 2 | 4.20 | 8/49 | 16.33 | 3.01±0.65 | yes |
| 3 | 5.64 | 6/46 | 13.04 | 3.25±1.28 | yes |
| 4 | 4.50 | 4/38 | 10.53 | 3.14±0.96 | yes |
| 5 | 4.68 | 12/47 | 25.53 | 3.24±0.87 | yes |
| 6 | 3.01 | 10/49 | 20.41 | 2.60±0.22 | no |
| 7 | 5.00 | 20/40 | 50.00 | 3.54±0.73 | yes |
| **Average** | **4.28** | **-** | **15.76** | **3.06** | **-** |
| SD (across participants) | 1.00 | - | 14.18 | 0.34 | - |
